# A CNP-cGMP-cGKI-MAPK pathway promotes melanoma growth in vitro and in vivo in mice

**DOI:** 10.1186/2050-6511-16-S1-A49

**Published:** 2015-09-02

**Authors:** Sandeep Dhayade, Susanne Feil, Anja Ulmer, Susanne Kaesler, Tobias Sinnberg, Martin Thunemann, Ulrike Naumann, Tilo Biedermann, Birgit Schittek, Robert Feil

**Affiliations:** 1 Interfakultäres Institut für Biochemie, University of Tübingen, Tübingen, Germany; 2 Hautklinik, University of Tübingen, Tübingen, Germany; 3 Hertie-Institut für Klinische Neurologie, Labor für Molekulare Neuroonkologie, Tübingen, Germany

## 

Several important effects of nitric oxide (NO) and natriuretic peptides (ANP, BNP, & CNP) are mediated by cyclic guanosine monophosphate (cGMP) and cGMP-dependent protein kinase type I (cGKI). Recent studies indicated that the cGMP pathway might also play a role in tumorigenesis. However, the cellular and molecular mechanisms of cGMP's effects on tumor growth are not well understood. In the present study, the expression and function of components of the cGMP pathway in melanoma cell lines of murine and human origin were investigated. We found that murine B16 melanoma cells express cGKI and treatment of the cells with the cell-permeable cGMP analog 8-bromo-cGMP induced the phosphorylation of cGKI substrate proteins VASP and PDE5. CNP, a ligand of the particulate guanylyl cyclase GC-B, but not NO or ANP, induced robust cGMP signals in melanoma cells. Interestingly, activation of the CNP-cGMP-cGKI pathway increased the growth and migration of B16 cells through a crosstalk with p44/42 MAPK signaling. The PDE5 inhibitor sildenafil enhanced CNP-mediated cGMP signaling and the growth of murine melanoma cells. Similar results were obtained with human melanoma cell lines. In line with the in vitro data, we found that cGKI overexpression in melanoma cells or administration of sildenafil promoted melanoma growth in vivo in mice. Finally, expression of cGKI was also detected in cancer cells of human melanoma patients. Taken together, we have identified a CNP-cGMP-cGKI-MAPK pathway in melanoma cells, which stimulates melanoma cell growth in vitro and in vivo and might be novel therapeutic target for treatment of melanoma.

